# Effect of Spatial Dispersion on Evolutionary Stability: A Two-Phenotype and Two-Patch Model

**DOI:** 10.1371/journal.pone.0142929

**Published:** 2015-11-13

**Authors:** Qing Li, Jiahua Zhang, Boyu Zhang, Ross Cressman, Yi Tao

**Affiliations:** 1 School of Mathematical Sciences, Capital Normal University, Beijing, China; 2 Key Lab of Animal Ecology and Conservational Biology, Institute of Zoology, Chinese Academy of Sciences, Beijing, China; 3 Laboratory of Mathematics and Complex Systems, Ministry of Education, School of Mathematical Sciences, Beijing Normal University, Beijing, China; 4 Department of Mathematics, Wilfrid Laurier University, Waterloo, ON, Canada; Shanxi University, CHINA

## Abstract

In this paper, we investigate a simple two-phenotype and two-patch model that incorporates both spatial dispersion and density effects in the evolutionary game dynamics. The migration rates from one patch to another are considered to be patch-dependent but independent of individual’s phenotype. Our main goal is to reveal the dynamical properties of the evolutionary game in a heterogeneous patchy environment. By analyzing the equilibria and their stabilities, we find that the dynamical behavior of the evolutionary game dynamics could be very complicated. Numerical analysis shows that the simple model can have twelve equilibria where four of them are stable. This implies that spatial dispersion can significantly complicate the evolutionary game, and the evolutionary outcome in a patchy environment should depend sensitively on the initial state of the patches.

## Introduction

In order to explain the evolution of animal behavior, Maynard Smith and Price [1] developed the concept of evolutionarily stable strategy (ESS) (see also [2–5]). Prior et al. [6] investigated an evolutionary game model that incorporates both spatial dispersion and density effects in the evolutionary dynamics. In this model, the population is considered to be dispersed in a patchy environment, where the background fitness and payoff matrix in each patch can be different. Migration from region to region is considered as an incidental aspect of the population, i.e., the migration is a chance event unrelated to an individual’s phenotype (strategy) or the fitness of the patch. As pointed out by Prior et al. [6], their assumptions differ from that of Ludwig and Levin [7] who treat the tendency to migrate as an individual characteristic subject to selection (see also [8–13]), and also differ from that of Hines and Maynard Smith [14] who interpret the effect of spatial dispersion as an increased tendency to interact with opponents sharing one’s own characteristics (see also [15]). Recently, Cressman and Krivan [16] investigated the migration dynamics for the ideal free distribution (IFD) in a patchy environment. They showed that IFD is evolutionarily stable under the assumptions that individuals never migrate from patches with a higher payoff to patches with a lower payoff and some individuals always migrate to the best patch. But migration does not necessarily lead to IFD if migration rates are independent of the payoffs of the patches.

For the evolutionary game dynamics in a patchy environment, Prior et al. [6] mainly focused their analysis on the stability of the homogeneous states, where they assumed that all patches have the same payoff matrix and density-dependent background fitness. Their main results showed that a stable equilibrium (e.g. an evolutionarily stable strategy) of the non-dispersed frequency dynamics becomes a stable equilibrium of the large system if population density stabilizes at these fixed frequencies.

In this paper, following Prior et al. [6], a simple two-patch and two-phenotype model is investigated. Three basic assumptions for this model are:

(i) The environment consists of two patches, called patch 1 and patch 2, respectively. Individuals can move from one patch to the other at any time *t*. The migration rates are patch-dependent but independent of individual’s phenotype [6]. Let *c*_1_ denote the probability that an individual moves from patch 1 to patch 2, and, similarly, *c*_2_ the probability that an individual moves from patch 2 to patch 1.
(ii) In each of two patches, individuals display two possible phenotypes (strategies), denoted by *R*_1_ and *R*_2_, and individuals interact in random pairwise contests. The payoff matrix is $\text{A}=(\begin{array}{cc} a_{11} & a_{12}\\ a_{21} & a_{22} \end{array})$ in patch 1 and $\text{B}=(\begin{array}{cc} b_{11} & b_{12}\\ b_{21} & b_{22} \end{array})$ in patch 2, where *a*_*ij*_ (or *b*_*ij*_) is the payoff of an individual displaying phenotype *R*_*i*_ when it plays against an individual displaying phenotype *R*_*j*_ in patch 1 (or in patch 2) for all *i*, *j* = 1, 2. Without loss of generality, we also assume that *a*_*ij*_ ≥ 0 and *b*_*ij*_ ≥ 0 for all *i*, *j* = 1, 2.
(iii) In each of the two patches, the background fitness is density-dependent [3–4], which is defined as *α*_1_ − *β*_1_ *n*_1_ in patch 1, and *α*_2_ − *β*_2_ *n*_2_ in patch 2, where *n*_1_ is the total population size in patch 1 and *n*_2_ the total population size in patch 2. We also assume that *α*_*i*_ > *c*_*i*_ for all *i* = 1, 2. That is, the migration rates are small enough to ensure that population size in a patch will increase when there are few individuals in the patch.

Let *x*_*i*_ denote the number of individuals with phenotype *R*_*i*_ in patch 1, and *y*_*i*_ the number of individuals with phenotype *R*_*i*_ in patch 2 (*i* = 1, 2). Clearly, *n*_1_ = *x*_1_ + *x*_2_ and *n*_2_ = *y*_1_ + *y*_2_. Similarly, let *p* denote the frequency of phenotype *R*_1_ in patch 1, and *q* the frequency of phenotype *R*_1_ in patch 2, i.e., *p* = *x*_1_/*n*_1_ and *q* = *y*_1_/*n*_2_. According to the basic assumption (ii), the expected payoff of an individual displaying phenotype *R*_*i*_ is *f*_*i*_ = *pa*_*i*1_ + (1 − *p*)*a*_*i*2_ in patch 1, and *g*_*i*_ = *qb*_*i*1_ + (1 − *q*)*b*_*i*2_ in patch 2 for *i* = 1, 2. Similarly, according to the basic assumption (iii), the (total) fitness of an individual displaying phenotype *R*_*i*_ is defined as *F*_*i*_ = (*α*_1_ − *β*_1_ *n*_1_) + *f*_*i*_ in patch 1, and *G*_*i*_ = (*α*_2_ − *β*_2_ *n*_2_) + *g*_*i*_ in patch 2 for *i* = 1, 2. Thus, the time evolution of *x*_*i*_ and *y*_*i*_ can be given by

$$\begin{array}{c} \begin{array}{cc} & \frac{dx_{i}}{dt}=\left[f_{i}+\left(\alpha_{1}-\beta_{1}n_{1}\right)\right]x_{i}-c_{1}x_{i}+c_{2}y_{i}\;,\\ & \frac{dy_{i}}{dt}=\left[g_{i}+\left(\alpha_{2}-\beta_{2}n_{2}\right)\right]y_{i}-c_{2}y_{i}+c_{1}x_{i}\;, \end{array} \end{array}$$ (1)

respectively, for *i* = 1, 2. Dynamics (1) also equivalent to the following system expressed in terms of phenotypic frequency and population size in each patch.

$$\begin{array}{c} \begin{array}{cc} & \frac{dp}{dt}=p\left(1-p\right)\left(f_{1}-f_{2}\right)+c_{2}\left(q-p\right)\frac{n_{2}}{n_{1}}\;,\\ & \frac{dq}{dt}=q\left(1-q\right)\left(g_{1}-g_{2}\right)+c_{1}\left(p-q\right)\frac{n_{1}}{n_{2}}\;,\\ & \frac{dn_{1}}{dt}=\left[\bar{f}+\left(\alpha_{1}-\beta_{1}n_{1}\right)\right]n_{1}-c_{1}n_{1}+c_{2}n_{2}\;,\\ & \frac{dn_{2}}{dt}=\left[\bar{g}+\left(\alpha_{2}-\beta_{2}n_{2}\right)\right]n_{2}-c_{2}n_{2}+c_{1}n_{1}\;, \end{array} \end{array}$$ (2)

where $\bar{f}=pf_{1}+\left(1-p\right)f_{2}$ and $\bar{g}=qg_{1}+\left(1-q\right)g_{2}$ are the average payoffs in patch 1 and patch 2, respectively.

In this paper, the equilibria of dynamics (2) and their stabilities are analyzed. Different from Prior et al. [6] who focused on the homogeneous states, we are primarily interested in analyzing the heterogeneous states, where two patches have different ESSs. Our main goal is to reveal the dynamical properties of the evolutionary game in a heterogeneous patchy environment.

## Results

### Symmetric equilibria of dynamics (2)

For given *p* and *q* with 0 ≤ *p*, *q* ≤ 1, an equilibrium of dynamics

$$\begin{array}{c} \begin{array}{cc} & \frac{dn_{1}}{dt}=n_{1}\;\left(\bar{f}+\left(\alpha_{1}-\beta_{1}n_{1}\right)\right)-c_{1}n_{1}+c_{2}n_{2}\;,\\ & \frac{dn_{2}}{dt}=n_{2}\;\left(\bar{g}+\left(\alpha_{2}-\beta_{2}n_{2}\right)\right)-c_{2}n_{2}+c_{1}n_{1}\;, \end{array} \end{array}$$ (3)

denoted by (*n*_1_(*p*, *q*), *n*_2_(*p*, *q*)), satisfies

$$\begin{array}{c} \begin{array}{cc} & n_{2}=\frac{n_{1}}{c_{2}}\left(c_{1}-\bar{f}-\left(\alpha_{1}-\beta_{1}n_{1}\right)\right)\;,\\ & n_{1}=\frac{n_{2}}{c_{1}}\left(c_{2}-\bar{g}-\left(\alpha_{2}-\beta_{2}n_{2}\right)\right)\;. \end{array} \end{array}$$ (4)

**Fig 1:**
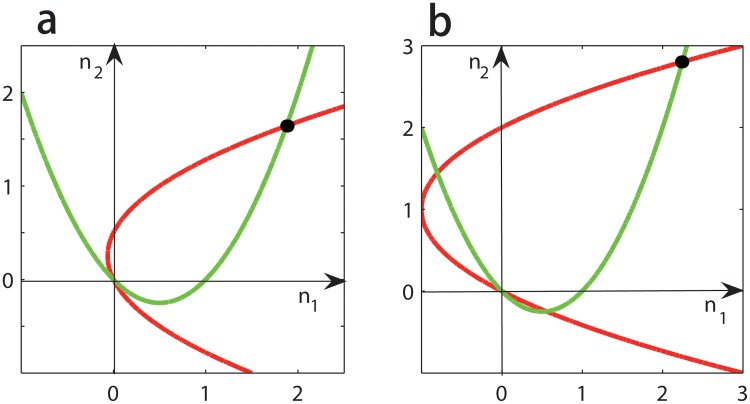
The unique positive equilibrium of dynamics (3). The red curves correspond to the second equation of Eq (4) and the green curves to the first equation of Eq (4). For any given *p* and *q*, the two curves have a unique positive intersection (see the black spots). Parameters are taken as *β*_1_ = *β*_2_ = 0.01, *α*_1_ = 0.75, $\bar{f}=0.25$, *c*_1_ = 0.5 and *c*_2_ = 0.5 in all two panels. Furthermore, *α*_2_ = 0.55 and $\bar{g}=0.2$ in panel **a**, and *α*_2_ = 1 and $\bar{g}=0.5$ in panel **b**.

It is clear that (*n*_1_, *n*_2_) = (0, 0) is always a solution of Eq (4) for any given *p* and *q*. Furthermore, (*p*, *q*, 0, 0) must be unstable under dynamics (2) since *α*_1_ > *c*_1_ and *α*_2_ > *c*_2_. Notice that *n*_2_ is a parabolic function of *n*_1_ and vice versa, Eq (4) also has a unique positive solution, denoted by $\left({\hat{n}}_{1},{\hat{n}}_{2})=(n_{1}\left(p,q\right),n_{2}\left(p,q\right)\right)$ with ${\hat{n}}_{1}>0$ and ${\hat{n}}_{2}>0$ (see Fig 1). When this solution corresponds to an equilibrium $\left(p,q,{\hat{n}}_{1},{\hat{n}}_{2}\right)$ of dynamics (2), we call it a *positive* equilibrium. In the rest of this paper, we only focus on the number and stabilities of these positive equilibria.

A positive equilibrium, $\left(p,q,{\hat{n}}_{1},{\hat{n}}_{2}\right)$, is called a *symmetric* equilibrium of dynamics (2) if *p* = *q*. That is, at a symmetric equilibrium, population compositions in the two patches are the same.

It is easy to see that dynamics (2) always have two symmetric boundary equilibria, $\left(1,1,{\hat{n}}_{1},{\hat{n}}_{2}\right)$ and $\left(0,0,{\hat{n}}_{1},{\hat{n}}_{2}\right)$, where at these equilibria, all individuals in the two patches display the same phenotype. Stabilities of the boundary equilibria can be characterized by analyzing the Jacobian matrix of dynamics (2) (see Method section). The main result is that if *R*_1_ (or *R*_2_) is an ESS for both payoff matrices **A** and **B**, then the boundary equilibrium $\left(1,1,{\hat{n}}_{1},{\hat{n}}_{2}\right)$ (or $\left(0,0,{\hat{n}}_{1},{\hat{n}}_{2}\right)$) must be asymptotically stable. This result is consistent with that of Prior et al. [6], where the stable equilibrium of the non-dispersed system becomes a stable equilibrium of dynamics (2). However, under the influence of migration, a symmetric boundary equilibria could be stable even if the corresponding phenotype is not an ESS in either patch. For instance, the boundary equilibrium $\left(1,1,{\hat{n}}_{1},{\hat{n}}_{2}\right)$ is asymptotically stable if *a*_11_ < *a*_21_ as long as *a*_21_ − *a*_11_ is small enough.

On the other hand, the symmetric interior equilibrium exists only for a very special case p* = q* ∈ (0, 1), where $p^{*}=\frac{a_{12}-a_{22}}{a_{12}-a_{22}+a_{21}-a_{11}}$ and $q^{*}=\frac{b_{12}-b_{22}}{b_{12}-b_{22}+b_{21}-b_{11}}$. In this case, $\left(p^{*},q^{*},{\hat{n}}_{1},{\hat{n}}_{2}\right)$ is an symmetric interior equilibrium of dynamics (2), where $\left({\hat{n}}_{1},{\hat{n}}_{2}\right)$ is the solution of Eq (4) for *p* = p* and *q* = q*. Furthermore, it is asymptotically stable if p* (= q*) is an ESS for both **A** and **B** (see Method section).

### General cases

For more general situations (i.e., p* ≠ q*), it is very tedious to determine the equilibria of dynamics (2). In fact, numerical simulations show that dynamics (2) may have twelve equilibria. To investigate the properties of the equilibria of dynamics (2), two cases are considered below. The first case is special in that there is no migration in one direction (i.e., one of *c*_1_ and *c*_2_ is 0) and we analyze the number of stable equilibria for all possible payoff structures. The second case is more general (i.e., *c*_1_ > 0 and *c*_2_ > 0) and we show the equilibria of dynamics (2) and their stabilities for 0 < p*, q* < 1.

**Case 1**. *c*_1_ > 0 **and** *c*_2_ = 0. Without loss of generality, we here assume that *c*_1_ > 0 but *c*_2_ = 0, i.e. individuals can only move from patch 1 to patch 2 but not from patch 2 to patch 1 (the case of *c*_1_ = 0 and *c*_2_ > 0 can be analyzed analogously). Then, dynamics (2) can be rewritten as

$$\begin{array}{c} \begin{array}{cc} & \frac{dp}{dt}=p\left(1-p\right)\left(f_{1}-f_{2}\right)\;,\\ & \frac{dn_{1}}{dt}=\left(\alpha_{1}+\bar{f}-c_{1}-\beta_{1}n_{1}\right)n_{1}\;, \end{array} \end{array}$$ (5)

and

$$\begin{array}{c} \begin{array}{cc} & \frac{dq}{dt}=q\left(1-q\right)\left(g_{1}-g_{2}\right)+c_{1}\left(p-q\right)\frac{n_{1}}{n_{2}},\\ & \frac{dn_{2}}{dt}=\left(\alpha_{2}+\bar{g}-\beta_{2}n_{2}\right)n_{2}+c_{1}n_{1}\;. \end{array} \end{array}$$ (6)

Notice that dynamics (5) is independent of dynamics (6). Thus, as an equilibrium of dynamics (2), $\left(\hat{p},\hat{q},{\hat{n}}_{1},{\hat{n}}_{2}\right)$, is locally asymptotically stable if and only if $\left(\hat{p},{\hat{n}}_{1}\right)$ is locally asymptotically stable under dynamics (5) and $\left(\hat{q},{\hat{n}}_{2}\right)$ is locally asymptotically stable under dynamics (6), where *p* and *n*_1_ in dynamics (6) correspond to the stable equilibrium, $\left(\hat{p},{\hat{n}}_{1}\right)$, of dynamics (5).

We first look at the stability of dynamics (5). It is easy to see that: (i) the boundary equilibrium $\left(1,{\hat{n}}_{1}\right)$ (or $\left(0,{\hat{n}}_{1}\right)$) is locally asymptotically stable if and only if *p* = 1 (or *p* = 0) is an ESS for the payoff matrix **A**, i.e. *a*_11_ > *a*_12_ (or *a*_22_ > *a*_12_), where ${\hat{n}}_{1}=\frac{\alpha_{1}+a_{11}-c_{1}}{\beta_{1}}$ for *p* = 1 and ${\hat{n}}_{1}=\frac{\alpha_{1}+a_{22}-c_{1}}{\beta_{1}}$ for *p* = 0; and (ii) if the unique interior equilibrium $\left(p^{*},{\hat{n}}_{1}\right)$ exists, then it is globally asymptotically stable if and only if p* is an ESS for the payoff matrix **A**, where $p^{*}=\frac{a_{12}-a_{22}}{a_{12}-a_{22}+a_{21}-a_{11}}\in \left(0,1\right)$ and ${\hat{n}}_{1}=\frac{\alpha_{1}+\bar{f}\left(p^{*}\right)-c_{1}}{\beta_{1}}$.

**Fig 2:**
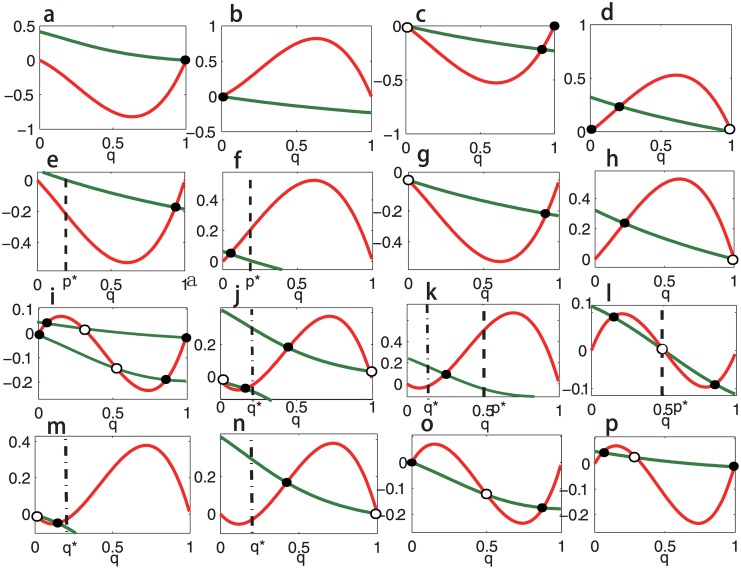
Solutions of Eq (7) and their stabilities under dynamics (2) when *c*_1_ > 0 and *c*_2_ = 0. Intersections of *h*_1_(*q*) and *h*_2_(*q*) (i.e., the solutions of Eq (7)) are shown for all sixteen possible situations. The red curves denote *h*_1_(*q*) and the green curves *h*_2_(*q*). The intersections denoted by black spots correspond to stable equilibria of dynamics (2), and the intersections denoted by black circles correspond to unstable equilibria. Parameters are taken as *β*_1_ = *β*_2_ = 0.01, *α*_1_ = 1, *α*_2_ = 2 and *c*_1_ = 0.5 in all sixteen panels. Payoff matrixes in the panels are: In panel **a**, **A** = [1.5, 1.5; 0, 0], **B** = [5, 1; 0, 0]. In panel **b**, **A** = [0, 0; 1, 1], **B** = [0, 0; 5, 1]. In panel **c**, **A** = [1, 0; 0, 1], **B** = [3, 1; 0, 0]. In panel **d**, **A** = [1, 0; 0, 1], **B** = [0, 0; 3, 1]. In panel **e**, **A** = [0, 1.25; 5, 0], **B** = [3, 1; 0, 0]. In panel **f**, **A** = [0, 1.25; 5, 0], **B** = [0, 0; 3, 1]. In panel **g**, **A** = [0, 0; 1, 1], **B** = [3, 1; 0, 0]. In panel **h**, **A** = [1, 1; 0, 0], **B** = [0, 0; 3, 1]. In panel **i**, **A** = [1, 0; 0, 1], **B** = [2, 0; 0, 1]. In panel **j**, **A** = [1, 0; 0, 1], **B** = [0, 1; 4, 0]. In panel **k**, **A** = [0, 2; 2, 0], **B** = [0, 1; 5, 0]. In panel **l**, **A** = [0, 2; 2, 0], **B** = [1, 0; 0, 1]. In panel **m**, **A** = [0, 0; 1, 1], **B** = [0, 1; 4, 0]. In panel **n**, **A** = [1.5, 1.5; 0, 0], **B** = [0, 1; 4, 0]. In panel **o**, **A** = [0, 0; 1, 1], **B** = [2, 0; 0, 1]. In panel **p**, **A** = [1, 1; 0, 0], **B** = [2, 0; 0, 1]. The positions of the interior ESSs p* and q* (0 < p*, q* < 1) are marked by dashed lines.

From dynamics (6), the frequency $\hat{q}$ in a stable equilibrium of dynamics (2), $\left(\hat{p},\hat{q},{\hat{n}}_{1},{\hat{n}}_{2}\right)$, should obey the equation

$$\begin{array}{ccc} q\left(1-q\right)\left(g_{1}-g_{2}\right)+\frac{\hat{p}-q}{2}\left[\sqrt{{\left(\alpha_{2}+\bar{g}\right)}^{2}+4\beta_{2}c_{1}{\hat{n}}_{1}}-\left(\alpha_{2}+\bar{g}\right)\right] & = & 0\;, \end{array}$$ (7)

where $\hat{p}\in \left\{0,1,p^{*}\right\}$ corresponds to the stable equilibrium, $\left(\hat{p},{\hat{n}}_{1}\right)$, of dynamics (5). In the Method section, we analyze the solutions of Eq (7) and the stabilities of the corresponding equilibria under dynamics (6). According to the stability conditions of dynamics (5) and (6), the equilibria of dynamics (2) and their properties can be summarized as follows:

1. If *R*_1_ (or *R*_2_) is the only ESS for **A** and **B**, then the symmetric boundary equilibrium $\left(0,0,{\hat{n}}_{1},{\hat{n}}_{2}\right)$ (or $\left(1,1,{\hat{n}}_{1},{\hat{n}}_{2}\right)$) is unstable and the other $\left(1,1,{\hat{n}}_{1},{\hat{n}}_{2}\right)$ (or $\left(0,0,{\hat{n}}_{1},{\hat{n}}_{2}\right)$) is stable. Furthermore, Eq (7) has no interior solution. This implies that $\left(1,1,{\hat{n}}_{1},{\hat{n}}_{2}\right)$ (or $\left(0,0,{\hat{n}}_{1},{\hat{n}}_{2}\right)$) is also globally asymptotically stable, i.e., all individuals in the two patches will eventually display *R*_1_ (or *R*_2_) under evolutionary dynamics (2) (see Fig 2A and 2B).
2. If both *R*_1_ and *R*_2_ are ESSs for **A** but *R*_1_ (or *R*_2_) is the only ESS for **B**, then the symmetric boundary equilibrium $\left(0,0,{\hat{n}}_{1},{\hat{n}}_{2}\right)$ (or $\left(1,1,{\hat{n}}_{1},{\hat{n}}_{2}\right)$) is unstable and the other $\left(1,1,{\hat{n}}_{1},{\hat{n}}_{2}\right)$ (or $\left(0,0,{\hat{n}}_{1},{\hat{n}}_{2}\right)$) is stable. Furthermore, Eq (7) has a unique (interior) solution $\hat{q}$, which corresponds to an asymptotically stable equilibrium $\left(0,\hat{q},{\hat{n}}_{1},{\hat{n}}_{2}\right)$ (or $\left(1,\hat{q},{\hat{n}}_{1},{\hat{n}}_{2}\right)$) of dynamics (2). In this situation, either all individuals in the system display *R*_1_ (or *R*_2_), or individuals in patch 1 display *R*_2_ (or *R*_1_) and two phenotypes coexist in patch 2 (see Fig 2C and 2D).
3. If p* is the only ESS for **A** (0 < p* < 1) and *R*_1_ (or *R*_2_) is the only ESS for **B**, then both the symmetric boundary equilibria $\left(0,0,{\hat{n}}_{1},{\hat{n}}_{2}\right)$ and $\left(1,1,{\hat{n}}_{1},{\hat{n}}_{2}\right)$ are unstable. Furthermore, Eq (7) has a unique (interior) solution $\hat{q}$, which corresponds to an asymptotically stable equilibrium $\left(p^{*},\hat{q},{\hat{n}}_{1},{\hat{n}}_{2}\right)$ of dynamics (2). Numerical simulation shows that this equilibrium is also globally stable. This implies that the two phenotypes will stably coexist in the system (see Fig 2E and 2F).
4. If *R*_2_ (or *R*_1_) is the only ESS for **A** but *R*_1_ (or *R*_2_) is the only ESS for **B**, then both the symmetric boundary equilibria $\left(0,0,{\hat{n}}_{1},{\hat{n}}_{2}\right)$ and $\left(1,1,{\hat{n}}_{1},{\hat{n}}_{2}\right)$ are unstable. Furthermore, Eq (7) has a unique (interior) solution $\hat{q}$, which corresponds to an asymptotically stable equilibrium $\left(0,\hat{q},{\hat{n}}_{1},{\hat{n}}_{2}\right)$ (or $\left(1,\hat{q},{\hat{n}}_{1},{\hat{n}}_{2}\right)$) of dynamics (2). Similarly as (**3**), this equilibrium is also globally stable, i.e., *R*_2_ (or *R*_1_) can invade patchy 2 under the influence of migration (see Fig 2G and 2H).
5. If both *R*_1_ and *R*_2_ are ESSs for **A** and **B**, then both the symmetric boundary equilibria $\left(0,0,{\hat{n}}_{1},{\hat{n}}_{2}\right)$ and $\left(1,1,{\hat{n}}_{1},{\hat{n}}_{2}\right)$ are asymptotically stable. Furthermore, Eq (7) has at most four (interior) solutions, where two are stable equilibria of dynamics (2) and the other two are unstable. This implies that the evolutionary outcome in this situation is very difficult to predict since the system can have four stable states (see Fig 2I).
6. If both *R*_1_ and *R*_2_ are ESSs for **A** and q* is an ESS for **B**, then both the symmetric boundary equilibria $\left(0,0,{\hat{n}}_{1},{\hat{n}}_{2}\right)$ and $\left(1,1,{\hat{n}}_{1},{\hat{n}}_{2}\right)$ are unstable. Furthermore, Eq (7) has at most two (interior) solutions, where both of them are stable equilibria of dynamics (2) (see Fig 2J).
7. If p* is an ESS for **A** and q* is an ESS for **B**, then both the symmetric boundary equilibria $\left(0,0,{\hat{n}}_{1},{\hat{n}}_{2}\right)$ and $\left(1,1,{\hat{n}}_{1},{\hat{n}}_{2}\right)$ are unstable. Furthermore, Eq (7) has a unique (interior) solution $\hat{q}$, which corresponds to an asymptotically stable equilibrium $\left(p^{*},\hat{q},{\hat{n}}_{1},{\hat{n}}_{2}\right)$ of dynamics (2). Similarly as (**3**), this equilibrium is also globally stable and two phenotypes will stably coexist in the system (see Fig 2K).
8. If p* is an ESS for **A** and both *R*_1_ and *R*_2_ are ESSs for **B**, then both the symmetric boundary equilibria $\left(0,0,{\hat{n}}_{1},{\hat{n}}_{2}\right)$ and $\left(1,1,{\hat{n}}_{1},{\hat{n}}_{2}\right)$ are unstable. Furthermore, Eq (7) has at most three (interior) solutions, which are denoted by ${\hat{q}}_{1}$, ${\hat{q}}_{2}$ and ${\hat{q}}_{3}$ with ${\hat{q}}_{1}<{\hat{q}}_{2}<{\hat{q}}_{3}$. The two interior equilibria corresponding to ${\hat{q}}_{1}$ and ${\hat{q}}_{3}$ are stable under dynamics (2) and the interior equilibrium corresponding to ${\hat{q}}_{2}$ is unstable (see Fig 2L).
9. If *R*_2_ (or *R*_1_) is the only ESS for **A** and q* is an ESS for **B**, then both the symmetric boundary equilibria $\left(0,0,{\hat{n}}_{1},{\hat{n}}_{2}\right)$ and $\left(1,1,{\hat{n}}_{1},{\hat{n}}_{2}\right)$ are unstable. Furthermore, Eq (7) has a unique (interior) solution $\hat{q}$, which corresponds to an asymptotically stable equilibrium $\left(0,\hat{q},{\hat{n}}_{1},{\hat{n}}_{2}\right)$ (or $\left(1,\hat{q},{\hat{n}}_{1},{\hat{n}}_{2}\right)$) of dynamics (2). Similarly as (**4**), this equilibrium is also globally stable and two phenotypes will stably coexist in patch 2 (see Fig 2M and 2N).
10. If *R*_2_ (or *R*_1_) is the only ESS for **A** and both *R*_1_ and *R*_2_ are ESSs for **B**, then the symmetric boundary equilibrium $\left(0,0,{\hat{n}}_{1},{\hat{n}}_{2}\right)$ (or $\left(1,1,{\hat{n}}_{1},{\hat{n}}_{2}\right)$) is stable and the other $\left(1,1,{\hat{n}}_{1},{\hat{n}}_{2}\right)$ (or $\left(0,0,{\hat{n}}_{1},{\hat{n}}_{2}\right)$) is unstable. Furthermore, Eq (7) has at most two (interior) solutions, where one corresponds to a stable equilibria of dynamics (2) and the other is unstable. Similarly as (**2**), either all individuals in the system display *R*_2_ (or *R*_1_), or individuals in patch 1 display *R*_2_ (or *R*_1_) and two phenotypes coexist in patch 2 (see Fig 2O and 2P).

**Case 2**. *c*_1_ > 0 **and** *c*_2_ > 0. We now consider the case with *c*_1_ > 0 and *c*_2_ > 0. It is easy to check that dynamics (2) only have two boundary equilibria, $\left(0,0,{\hat{n}}_{1},{\hat{n}}_{2}\right)$ and $\left(1,1,{\hat{n}}_{1},{\hat{n}}_{2}\right)$, and the existence of asymmetric boundary equilibrium is impossible, for instance, if *p* = 0 and *q* ≠ 0, then $\frac{dp}{dt}>0$. We then focus on the number and stability of interior equilibria. Notice that an equilibrium of dynamics (2) should be the solution of equation

$$\begin{array}{c} \begin{array}{cc} & \Delta_{1}p\left(1-p\right)\left(p-p^{*}\right)+c_{2}\left(q-p\right)\frac{n_{2}}{n_{1}}\;=\;0\;,\\ & \Delta_{2}q\left(1-q\right)\left(q-q^{*}\right)+c_{1}\left(p-q\right)\frac{n_{1}}{n_{2}}\;=\;0\;,\\ & \left[\bar{f}+\left(\alpha_{1}-\beta_{1}n_{1}\right)\right]n_{1}-c_{1}n_{1}+c_{2}n_{2}\;=\;0\;,\\ & \left[\bar{g}+\left(\alpha_{2}-\beta_{2}n_{2}\right)\right]n_{2}-c_{2}n_{2}+c_{1}n_{1}\;=\;0\;. \end{array} \end{array}$$ (8)

So if both *n*_1_ and *n*_2_ are positive, then from the first two equations of Eq (8), an interior equilibrium of dynamics (2) should obey the equations

$$\begin{array}{c} \Delta_{1}\Delta_{2}p\left(1-p\right)q\left(1-q\right)\left(p-p^{*}\right)\left(q-q^{*}\right)\;=\;-c_{1}c_{2}{\left(p-q\right)}^{2}. \end{array}$$ (9)

Furthermore, from the third and the forth equations of Eq (8)

$$\begin{array}{c} \frac{\Delta_{1}p\left(1-p\right)\left(p-p^{*}\right)}{c_{2}\left(p-q\right)}\;=\;\frac{\beta_{1}}{\beta_{2}}\cdot \frac{\bar{g}+\alpha_{2}-c_{2}+\frac{\Delta_{2}q\left(1-q\right)\left(q-q^{*}\right)}{q-p}}{\bar{f}+\alpha_{1}-c_{1}+\frac{\Delta_{1}p\left(1-p\right)\left(p-p^{*}\right)}{p-q}}, \end{array}$$ (10)

where we assume that both p* and q* are in the interval 0 < p*, q* < 1 (i.e., we consider the most complicated payoff structures).

**Fig 3:**
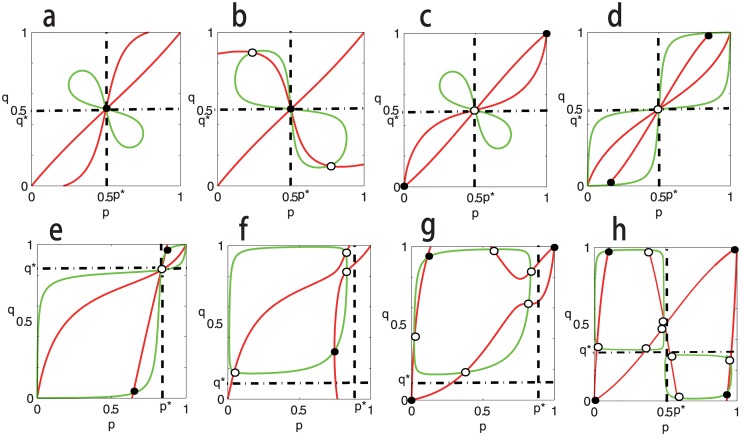
Equilibria of Eq (2) on the *p* − *q* plane and their stabilities when *c*_1_ > 0 and *c*_2_ > 0. The red curves correspond to Eq (10) and the green curves Eq (9). The intersections denoted by black spot correspond to stable equilibria of dynamics (2), and the intersections denoted by black circle correspond to unstable equilibria. Parameters are taken as: In panel **a**, **A** = [0, 1; 1, 0], **B** = [0, 5; 5, 0], *β*_1_ = *β*_2_ = 0.01, *α*_1_ = 1.25, *α*_2_ = 1.8, *c*_1_ = 0.25 and *c*_2_ = 0.8. In panel **b**, **A** = [0, 1; 1, 0], **B** = [0, 5; 5, 0], *β*_1_ = *β*_2_ = 0.01, *α*_1_ = 1.125, *α*_2_ = 0.1, *c*_1_ = 0.125 and *c*_2_ = 0.8. In panel **c**, **A** = [1, 0; 0, 1], **B** = [5, 0; 0, 5], *β*_1_ = *β*_2_ = 0.001, *α*_1_ = 1.4, *α*_2_ = 1.5, *c*_1_ = 0.4 and *c*_2_ = 0.5. In panel **d**, **A** = [0, 1; 1, 0], **B** = [5, 0; 0, 5] *β*_1_ = *β*_2_ = 0.001, *α*_1_ = 1.5, *α*_2_ = 1.2, *c*_1_ = 0.5 and *c*_2_ = 0.2. In panel **e**, **A** = [0, 5; 1, 0], **B** = [1, 0; 0, 5], *β*_1_ = *β*_2_ = 0.001, *α*_1_ = 1.5, *α*_2_ = 1.2, *c*_1_ = 0.5 and *c*_2_ = 0.2. In panel **f**, **A** = [0, 5; 1, 0], **B** = [0, 1; 5, 0], *β*_1_ = *β*_2_ = 0.001, *α*_1_ = 1.5, *α*_2_ = 1.2, *c*_1_ = 0.5 and *c*_2_ = 0.2. In panel **g**, **A** = [1, 0; 0, 5], **B** = [5, 0; 0, 1], *β*_1_ = *β*_2_ = 0.001, *α*_1_ = 1.4, *α*_2_ = 1.5, *c*_1_ = 0.4 and *c*_2_ = 0.5. In panel **h**, **A** = [1, 0; 0, 1], **B** = [2, 0; 0, 1], *β*_1_ = 0.05, *β*_2_ = 0.01, *α*_1_ = 1.75, *α*_2_ = 2, *c*_1_ = 0.25 and *c*_2_ = 0.01. The positions of p* and q* are marked by dashed lines (0 < p*, q* < 1, and note that p* and q* may not be ESS).

From Eq (9), it is easy to see that for the situation with p* ≠ q*, if the interior equilibrium exists, then it should be in the region (0, p*) × (q*, 1), or (p*, 1) × (0, q*) if Δ_1_Δ_2_ > 0, and in the region (0, p*) × (0, q*), or (p*, 1) × (q*, 1) if Δ_1_Δ_2_ < 0. Of course, it is very difficult to get the exactly analytic solutions of Eqs (9) and (10) in general. The numerical analysis suggests that ten interior equilibria can exist (see Fig 3H). To show this, some examples are plotted in Fig 3. All of these examples show clearly that the equilibrium structure of dynamics (2) could be very complicated.

**Fig 4:**
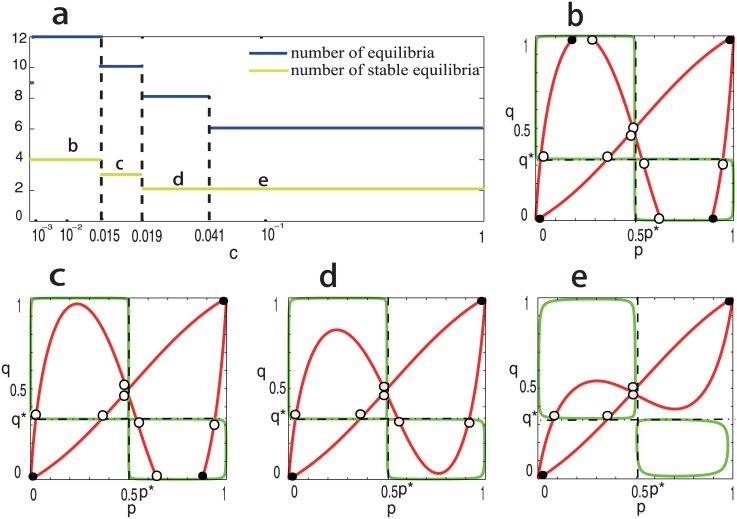
Bifurcation behaviors of Eq (2) when *c*_1_ > 0 and *c*_2_ > 0. In panel **a**, the number of equilibria and the number of stable equilibria are denoted by blue line and yellow line, respectively. In panels **b-e**, the red curves correspond to Eq (10) and the green curves Eq (9). The intersections denoted by black spot correspond to stable equilibria of dynamics (2), and the intersections denoted by black circle correspond to unstable equilibria. The positions of p* and q* are marked by dashed lines. Parameters are taken as: **A** = [1, 0; 0, 1], **B** = [2, 0; 0, 1], *β*_1_ = 0.05, *β*_2_ = 0.01, *α*_1_ = 1.75, *α*_2_ = 2. In panel **b**, *c*_1_ = *c*_2_ = *c* = 0.014; in panel **c**, *c*_1_ = *c*_2_ = *c* = 0.015; in panel **d**, *c*_1_ = *c*_2_ = *c* = 0.02; and in panel **e**, *c*_1_ = *c*_2_ = *c* = 0.05.

We further look at the bifurcation behaviors of system (2) for the case that both *R*_1_ and *R*_2_ are ESSs for **A** and **B** (i.e., the most complicated case), and assume equal migration rates between regions, i.e., *c*_1_ = *c*_2_ = *c*. In this case, both the symmetric boundary equilibria $\left(0,0,{\hat{n}}_{1},{\hat{n}}_{2}\right)$ and $\left(1,1,{\hat{n}}_{1},{\hat{n}}_{2}\right)$ are asymptotically stable, and the system can have ten interior equilibria. When *c* = 0, it is easy to see that the system has nine equilibria in total, including four stable (boundary) equilibria and five unstable equilibria. The number of equilibria jumps from nine to twelve as soon as the migration rates becomes positive although the number of stable equilibria keeps unchanged (see Fig 4A and 4B). Furthermore, numerical simulation shows that both the numbers of stable equilibria and unstable equilibria decrease as *c* increases. In particular, when *c* > 0.019, the system has only two stable equilibria (i.e., the two symmetric boundary equilibria), and all interior equilibria are unstable (see Fig 4). These results suggest that small migration rates make the dynamical behavior of the system more complex.

## Discussion

A vast amount of research has been devoted to analyze the influence of spatial diffusion on the evolutionary stability of ecology systems. One well known mathematical approach is the reaction-diffusion equation, where in this framework, individuals are dispersed in a continues space [10–11, 17–21]. For instance, Hofbauer et al. [11, 21] considered a population of two types of individuals distributed in an one-dimensional space, and assumed that the migration (or diffusion) rate is both individual-independent and location-independent. They showed that in two-strategy coordination games, if the reaction term of the reaction-diffusion equation is taken as replicator dynamics, then one strategy will drive out the other strategy in form of a traveling wave front, although there is no simple rule to decide which strategy can survive.

In this paper, we assume that individuals are distributed in a (discrete) patchy environment. Following Prior et al. [6], we investigate a simple two-phenotype and two-patch model, where individuals compete only with their immediate neighbors and the migration rates between patches are individual-independent but patch-dependent. Different from Prior et al. [6] who focused on the homogeneous patchy environment, we here are more interested in the dynamical stability in heterogeneous environment. Our main results show that: (i) if the pure strategy *R*_1_ (or *R*_2_) is an ESS for both two patches, then the boundary equilibrium corresponding to *p* = 1 and *q* = 1 (or *p* = 0 and *q* = 0) must be asymptotically stable; (ii) if the payoff matrices **A** and **B** satisfy $p^{*}=\frac{a_{12}-a_{22}}{a_{12}-a_{22}+a_{21}-a_{11}}=q^{*}=\frac{b_{12}-b_{22}}{b_{12}-b_{22}+b_{21}-b_{11}}\in \left(0,1\right)$, then the interior equilibrium corresponding to (p*, q*) is asymptotically stable if p* is an ESS for **A** and q* an ESS for **B**; (iii) as a special case with *c*_1_ > 0 and *c*_2_ = 0 (or *c*_1_ = 0 and *c*_2_ > 0), i.e. individuals can only move from patch 1 to patch 2 (or from patch 2 to patch 1), all possible situations for the existence and stability of boundary and interior equilibria are considered, and we find that dynamics (2) can have six equilibria where four of them are stable; (iv) for *c*_1_ > 0 and *c*_2_ > 0, the numerical analysis shows that the equilibrium structure and dynamical behavior of the system could be very complicated in general. In particular, dynamics (2) can have twelve equilibria where four of them are stable.

Our analysis provides an insight for understanding the effect of spatial dispersion on the evolutionary stability of patchy environment. Both the analytical analysis and the numerical simulation indicate that the original ESS formulations which ignore the dispersion process cannot be applied to predict the evolutionary outcome of the dispersion system even for small migration rates. For instance, in the case that both patches have multiple ESS’s and no dispersal between patches, the system has four (boundary) stable equilibria and five unstable equilibria. However, if one of *c*_1_ and *c*_2_ becomes positive, the system can have two to four stable equilibria and four to eight unstable equilibria. Furthermore, we found that both the numbers of stable equilibria and unstable equilibria decrease in the migration rates. This observation has an intuitive biological interpretation [6]. In a heterogenous patchy environment, the effect of selection is to make the overall population more heterogeneous in the sense of different patches have different population compositions, while the effect of migration is to move the population composition in each patch towards the mean of the overall population, i.e., migration promotes homogeneity. Thus, when the migration rates are small (i.e., the effect of selection is strong), similarly as the case of no dispersal, the system has two stable symmetric boundary equilibria and two stable asymmetric equilibria; and when the migration rates are large (i.e., the effect of migration is strong), the existence of stable asymmetric equilibrium is impossible, and the system has only two stable symmetric boundary equilibria, where at these equilibria all individuals display the same phenotype.

In this paper, we focus on the effect of spatial dispersion on two-patch system only. A natural extension would be to consider the three-patch system. However, analyzing the dynamical behavior of the three-patch system may be an even more difficult issue because the equilibrium structure of the two-patch system is already very complex. Another possible development would be to compare the evolutionary stability of the patchy environment under different migration rules. One commonly used migration rule is that individuals know perfectly the payoff in all patches and they always move to the patch with the highest payoff (i.e., ideal animals) [15]. In contrast, a more realistic model is that individuals do not migrate to patches with lower payoff [22]. Recent studies have shown that these two migration rules can lead to the IFD [16, 23]. Since that the IFD corresponds to a stable equilibrium of the non-dispersed evolutionary dynamics, we can then expect that these migration rules may also lead to the ESS of the non-dispersed evolutionary dynamics [23].

## Methods

### Stability of the symmetric equilibria

The Jacobian matrix of the dynamics (2) about the symmetric boundary equilibrium $\left(1,1,{\hat{n}}_{1},{\hat{n}}_{2}\right)$, denoted by **J**_(1,1)_, is

$$\begin{array}{c} \left(\begin{array}{cccc} -\left(a_{11}-a_{21}\right)-c_{2}\frac{{\hat{n}}_{2}}{{\hat{n}}_{1}} & c_{2}\frac{{\hat{n}}_{2}}{{\hat{n}}_{1}} & 0 & 0\\ c_{1}\frac{{\hat{n}}_{1}}{{\hat{n}}_{2}} & -\left(b_{11}-b_{21}\right)-c_{1}\frac{{\hat{n}}_{1}}{{\hat{n}}_{2}} & 0 & 0\\ \left(2a_{11}-a_{12}-a_{21}\right){\hat{n}}_{1} & 0 & -\beta_{1}{\hat{n}}_{1}-c_{2}\frac{{\hat{n}}_{2}}{{\hat{n}}_{1}} & c_{2}\\ 0 & \left(2b_{11}-b_{12}-b_{21}\right){\hat{n}}_{2} & c_{1} & -\beta_{2}{\hat{n}}_{2}-c_{1}\frac{{\hat{n}}_{1}}{{\hat{n}}_{2}} \end{array}\right)\;, \end{array}$$

and similarly, the Jacobian matrix about $\left(0,0,{\hat{n}}_{1},{\hat{n}}_{2}\right)$, denoted by **J**_(0,0)_, is

$$\begin{array}{c} \left(\begin{array}{cccc} \left(a_{12}-a_{22}\right)-c_{2}\frac{{\hat{n}}_{2}}{{\hat{n}}_{1}} & c_{2}\frac{{\hat{n}}_{2}}{{\hat{n}}_{1}} & 0 & 0\\ c_{1}\frac{{\hat{n}}_{1}}{{\hat{n}}_{2}} & \left(b_{12}-b_{22}\right)-c_{1}\frac{{\hat{n}}_{1}}{{\hat{n}}_{2}} & 0 & 0\\ \left(a_{12}+a_{21}-2a_{22}\right){\hat{n}}_{1} & 0 & -\beta_{1}{\hat{n}}_{1}-c_{2}\frac{{\hat{n}}_{2}}{{\hat{n}}_{1}} & c_{2}\\ 0 & \left(b_{12}+b_{21}-2b_{22}\right){\hat{n}}_{2} & c_{1} & -\beta_{2}{\hat{n}}_{2}-c_{1}\frac{{\hat{n}}_{1}}{{\hat{n}}_{2}} \end{array}\right)\;. \end{array}$$

For the matrix **J**_(1,1)_, notice that the eigenvalues of the matrix

$$\begin{array}{c} \left(\begin{array}{cc} -\left(a_{11}-a_{21}\right)-c_{2}\frac{{\hat{n}}_{2}}{{\hat{n}}_{1}} & c_{2}\frac{{\hat{n}}_{2}}{{\hat{n}}_{1}}\\ c_{1}\frac{{\hat{n}}_{1}}{{\hat{n}}_{2}} & -\left(b_{11}-b_{21}\right)-c_{1}\frac{{\hat{n}}_{1}}{{\hat{n}}_{2}} \end{array}\right) \end{array}$$

have negative real parts if *a*_11_ − *a*_21_ > 0 and *b*_11_ − *b*_21_ > 0, and that the real parts of the eigenvalues of the matrix

$$\begin{array}{c} \left(\begin{array}{cc} -\beta_{1}{\hat{n}}_{1}-c_{2}\frac{{\hat{n}}_{2}}{{\hat{n}}_{1}} & c_{2}\\ c_{1} & -\beta_{2}{\hat{n}}_{2}-c_{1}\frac{{\hat{n}}_{1}}{{\hat{n}}_{2}} \end{array}\right) \end{array}$$

must be negative. So, if the pure strategy *R*_1_ is an ESS for both payoff matrices **A** and **B**, then the eigenvalues of **J**_(1,1)_ must have negative real parts [6]. Similar to the matrix **J**_(0,0)_, if the pure strategy *R*_2_ is an ESS for both payoff matrices **A** and **B**, then the eigenvalues of **J**_(0,0)_ have negative real parts.

The Jacobian matrix about the symmetric interior equilibrium $\left(p^{*},q^{*},{\hat{n}}_{1},{\hat{n}}_{2}\right)$, denoted by **J**_(p*, q*)_, is

$$\begin{array}{c} \left(\begin{array}{cccc} p^{*}\left(1-p^{*}\right)\Delta_{1}-c_{2}\frac{{\hat{n}}_{2}}{{\hat{n}}_{1}} & c_{2}\frac{{\hat{n}}_{2}}{{\hat{n}}_{1}} & 0 & 0\\ c_{1}\frac{{\hat{n}}_{1}}{{\hat{n}}_{2}} & q^{*}\left(1-q^{*}\right)\Delta_{2}-c_{1}\frac{{\hat{n}}_{1}}{{\hat{n}}_{2}} & 0 & 0\\ \left(-a_{12}+a_{21}\right){\hat{n}}_{1} & 0 & -\beta_{1}{\hat{n}}_{1}-c_{2}\frac{{\hat{n}}_{2}}{{\hat{n}}_{1}} & c_{2}\\ 0 & \left(-b_{12}+b_{21}\right){\hat{n}}_{2} & c_{1} & -\beta_{2}{\hat{n}}_{2}-c_{1}\frac{{\hat{n}}_{1}}{{\hat{n}}_{2}} \end{array}\right)\;, \end{array}$$

where Δ_1_ = *a*_11_ − *a*_12_ − *a*_21_ + *a*_22_ and Δ_2_ = *b*_11_ − *b*_12_ − *b*_21_ + *b*_22_. Also similar to the matrix **J**_(1,1)_ (or the matrix **J**_(0,0)_), the eigenvalues of **J**_(p*, q*)_ have the negative real parts if p* (= q*) is an ESS for both payoff matrices **A** and **B**, i.e., the equilibrium $\left(p^{*},q^{*},{\hat{n}}_{1},{\hat{n}}_{2}\right)$ is asymptotically stable if p* (= q*) is an ESS for both **A** and **B**.

### Stability analysis of dynamics (6) when *c*_1_ > 0 and *c*_2_ = 0

We first analyze the solutions of Eq (7). For convenience, let

$$\begin{array}{ccc} h_{1}\left(q\right) & = & -\Delta_{2}q\left(1-q\right)\left(q-q^{*}\right)\;,\\ h_{2}\left(q\right) & = & \frac{\hat{p}-q}{2}\;\left[\sqrt{{\left(\alpha_{2}+\bar{g}\right)}^{2}+4\beta_{2}c_{1}{\hat{n}}_{1}}-\left(\alpha_{2}+\bar{g}\right)\right]\;. \end{array}$$

It is easy to see that Eq (7) has a boundary solution $\hat{q}=0$ (or *q* = 1) if and only if $\hat{p}=0$ (or $\hat{p}=1$). Furthermore, the interior solutions of Eq (7) should correspond to the intersections of the functions *h*_1_(*q*) and *h*_2_(*q*) in the interval 0 < *q* < 1. Notice that *h*_1_(0) = *h*_1_(1) = *h*_1_(q*) = 0, Δ_2_ *h*_1_(*q*) > 0 for 0 < *q* < q* and Δ_2_ *h*_1_(*q*) < 0 for q* < *q* < 1 (if 0 < q* < 1), and that *h*_2_(0) ≥ 0, *h*_2_(1) ≤ 0, $h_{2}\left(\hat{p}\right)=0$, *h*_2_(*q*) > 0 for $0<q<\hat{p}$ and *h*_2_(*q*) < 0 for $\hat{p}<q<1$ (if $0<\hat{p}<1$). Then, for the existence of intersections in the interval 0 < *q* < 1, we have that:

1. If *R*_1_ is the only ESS for both payoff matrices **A** and **B**, then, no intersection can exist (see Fig 2A); and, similarly, if *R*_2_ is the only ESS for both **A** and **B**, then no intersection can exist (see Fig 2B).
2. If both *R*_1_ and *R*_2_ are ESSs for **A** but *R*_1_ is the only ESS for **B**, then only one intersection exists (see Fig 2C); and, similarly, if both *R*_1_ and *R*_2_ are ESSs for **A** but *R*_2_ is the only ESS for **B**, then only one intersection exists (see Fig 2D).
3. If p* ∈ (0, 1) is an ESS for **A** and *R*_1_ is the only ESS for **B**, only one intersection exists (see Fig 2E); and, similarly, if p* is an ESS for **A** and *R*_2_ is the only ESS for **B**, then only one intersection exists (Fig 2F).
4. If *R*_2_ is the only ESS for **A** and *R*_1_ is the only ESS for **B**, then only one intersection exists (see Fig 2G); and, similarly, if *R*_1_ is the only ESS for **A** and *R*_2_ is the only ESS for **B**, then only one intersection exists (see Fig 2H).
5. If both *R*_1_ and *R*_2_ are ESSs for **A** and **B**, then at most four intersections can exist (see Fig 2I).
6. If both *R*_1_ and *R*_2_ are ESSs for **A** and q* ∈ (0, 1) is an ESS for **B**, only two intersections exist (see Fig 2J).
7. If p* is an ESS for **A** and q* is an ESS for **B**, the only one intersection exists (see Fig 2K).
8. If p* is an ESS for **A** and both *R*_1_ and *R*_2_ are ESSs for **B**, then there are at most three intersections (see Fig 2L).
9. If *R*_2_ is the only ESS for **A** and q* is an ESS for **B**, then only one intersection exists (see Fig 2M); and, similarly, If *R*_1_ is the only ESS for **A** and q* is an ESS for **B**, then only one intersection exists (see Fig 2N).
10. If *R*_2_ is the only ESS for **A** and both *R*_1_ and *R*_2_ are ESSs for **B**, then there are at most two intersections (see Fig 2O); and, similarly, if *R*_1_ is the only ESS for **A** and both *R*_1_ and *R*_2_ are ESSs, then there are at most two intersections (see Fig 2P).

For the stability of the solutions of Eq (7) under dynamics (6), it is easy to see that for given $\hat{p}$ (i.e. $\hat{p}\in \left\{0,1,p^{*}\right\}$ corresponds to the stable equilibrium of dynamics (5)), if $\tilde{q}=\hat{p}$ and $\tilde{q}$ is an ESS for the payoff matrix **B**, then the corresponding equilibrium $\left(\tilde{q},{\hat{n}}_{2}\right)$ must be asymptotically stable under dynamics (6). On the hand, let $\left(\hat{q},{\hat{n}}_{2}\right)$ be an interior equilibrium of dynamics (6), and the Jacobian matrix about $\left(\hat{q},{\hat{n}}_{2}\right)$, denoted by $J_{\left(\hat{q},{\hat{n}}_{2}\right)}$, is given by

$$\begin{array}{ccc} J_{\left(\hat{q},{\hat{n}}_{2}\right)} & = & \left(\begin{array}{cc} -\frac{h_{1}\left(q\right)}{dq}{|}_{q=\hat{q}}-c_{1}\frac{{\hat{n}}_{1}}{{\hat{n}}_{2}} & -\left(\hat{p}-\hat{q}\right)c_{1}\frac{{\hat{n}}_{1}}{{\hat{n}}_{2}^{2}}\\ \frac{d\bar{g}\left(q\right)}{dq}{|}_{q=\hat{q}}{\hat{n}}_{2} & -c_{1}\frac{{\hat{n}}_{1}}{{\hat{n}}_{2}}-\beta_{2}{\hat{n}}_{2} \end{array}\right)\;. \end{array}$$

Clearly, the interior equilibrium $\left(\hat{q},{\hat{n}}_{2}\right)$ is asymptotically stable (i.e. the eigenvalues of $J_{\left(\hat{q},{\hat{n}}_{2}\right)}$ have the negative real parts) if

$$\begin{array}{ccc} & & -\frac{h_{1}\left(q\right)}{dq}{|}_{q=\hat{q}}-2c_{1}\frac{{\hat{n}}_{1}}{{\hat{n}}_{2}}-\beta_{2}{\hat{n}}_{2}\;<\;0\;,\\ & & \left(\frac{h_{1}\left(q\right)}{dq}{|}_{q=\hat{q}}+c_{1}\frac{{\hat{n}}_{1}}{{\hat{n}}_{2}}\right)\left(c_{1}\frac{{\hat{n}}_{1}}{{\hat{n}}_{2}}+\beta_{2}{\hat{n}}_{2}\right)+\left(\hat{p}-\hat{q}\right)c_{1}\frac{{\hat{n}}_{1}}{{\hat{n}}_{2}}\cdot \frac{d\bar{g}\left(q\right)}{dq}{|}_{q=\hat{q}}\;>\;0\;. \end{array}$$

Thus, for given parameter values, stabilities of the interior equilibria of dynamics (6) (i.e., interior solutions of Eq (7)) can be analyzed numerically according to the above conditions (see the figure caption of Fig 2 for detailed parameters, note that the following results may not be true for all parameter values).

1. If *R*_1_ (or *R*_2_) is the only ESS for **A** and **B**, then the boundary equilibrium $\left(1,{\hat{n}}_{2}\right)$ with $\hat{p}=1$ (or $\left(0,{\hat{n}}_{2}\right)$ with $\hat{p}=0$) is asymptotically stable (see also Fig 2A and 2B).
2. If both *R*_1_ and *R*_2_ are ESSs for **A** but *R*_1_ (or *R*_2_) is the only ESS for **B**, then one boundary equilibrium $\left(0,{\hat{n}}_{2}\right)$ with $\hat{p}=0$ (or $\left(1,{\hat{n}}_{2}\right)$ with $\hat{p}=1$) is unstable and the other boundary equilibrium $\left(1,{\hat{n}}_{2}\right)$ with $\hat{p}=1$ (or $\left(0,{\hat{n}}_{2}\right)$ with $\hat{p}=0$) is asymptotically stable. Furthermore, the unique interior equilibrium $\left(\hat{q},{\hat{n}}_{2}\right)$ is also asymptotically stable (see also Fig 2C and 2D).
3. If p* is an ESS for **A** and *R*_1_ (or *R*_2_) is the only ESS for **B**, then the unique interior equilibrium is asymptotically stable (see also Fig 2E and 2F).
4. If *R*_2_ (or *R*_1_) is the only ESS for **A** but *R*_1_ (or *R*_2_) is the only ESS for **B**, then the boundary equilibrium $\left(0,{\hat{n}}_{2}\right)$ with $\hat{p}=0$ (or $\left(1,{\hat{n}}_{2}\right)$ with $\hat{p}=1$) is unstable and the unique interior equilibrium is asymptotically stable (see also Fig 2G and 2H).
5. If both *R*_1_ and *R*_2_ are ESSs for **A** and **B**, then the boundary equilibrium $\left(1,{\hat{n}}_{2}\right)$ with $\hat{p}=1$, or the boundary equilibrium $\left(0,{\hat{n}}_{2}\right)$ with $\hat{p}=0$, is stable, and for four interior equilibria, two are stable and the other two are unstable (see also Fig 2I).
6. If both *R*_1_ and *R*_2_ are ESSs for **A** and q* is an ESS for **B**, then the boundary equilibrium $\left(1,{\hat{n}}_{2}\right)$ with $\hat{p}=1$, or the boundary equilibrium $\left(0,{\hat{n}}_{2}\right)$ with $\hat{p}=0$, is unstable, and the two interior equilibria are asymptotically stable (see also Fig 2J).
7. If p* is an ESS for **A** and q* is an ESS for **B**, then the unique interior equilibrium is asymptotically stable (see also Fig 2K).
8. If p* is an ESS for **A** and both *R*_1_ and *R*_2_ are ESSs for **B**, then there are at most three interior equilibria corresponding to three intersections of *h*_1_ and *h*_2_, which are denoted by ${\hat{q}}_{1}$, ${\hat{q}}_{2}$ and ${\hat{q}}_{3}$ with ${\hat{q}}_{1}<{\hat{q}}_{2}<{\hat{q}}_{3}$, the two interior equilibria corresponding to ${\hat{q}}_{1}$ and ${\hat{q}}_{3}$, respectively, are stable and the interior equilibrium corresponding to ${\hat{q}}_{2}$ is unstable (see also Fig 2L).
9. If *R*_2_ (or *R*_1_) is the only ESS for **A** and q* is an ESS for **B**, then the boundary equilibrium $\left(0,{\hat{n}}_{2}\right)$ with $\hat{p}=0$ (or $\left(1,{\hat{n}}_{2}\right)$ with $\hat{p}=1)$ is unstable and the unique interior equilibrium is asymptotically stable (see also Fig 2M and 2N).
10. If *R*_2_ (or *R*_1_) is the only ESS for **A** and both *R*_1_ and *R*_2_ are ESSs for **B**, then there are at most two interior equilibria, the boundary equilibrium $\left(0,{\hat{n}}_{2}\right)$ for $\hat{p}=0$ (or $\left(1,{\hat{n}}_{2}\right)$ for $\hat{p}=1$) is stable, and one interior equilibrium is stable and the other unstable (see also Fig 2O and 2P).
